# Severe Renal Hemorrhage Following Nephrostomy Tube Removal: A Case Report

**DOI:** 10.7759/cureus.117390

**Published:** 2026-10-03

**Authors:** Yoshihiko Ogata, Masato Yanagi, Yoshihiro Nishikawa, Kuniaki Tanabe, Shuichi Osawa, Jun Akatsuka

**Affiliations:** 1 Urology, Nippon Medical School Hospital, Tokyo, JPN; 2 Urology, Heisei Tateishi Hospital, Tokyo, JPN

**Keywords:** hematuria, ileal conduit, nephrostomy tube removal, percutaneous nephrostomy, renal hemorrhage

## Abstract

Percutaneous nephrostomy (PCN) is widely performed to drain upper urinary tract obstructions. Although hemorrhagic complications during PCN placement are well recognized, clinically significant bleeding immediately following nephrostomy tube removal has been rarely reported. A 76-year-old man with muscle-invasive bladder cancer and bilateral hydronephrosis underwent right-sided PCN for obstructive pyelonephritis using an 8.3-Fr JINRO™ nephrostomy catheter (Boston Scientific, Marlborough, MA, USA). He subsequently underwent radical cystectomy, pelvic lymph node dissection, and ileal conduit urinary diversion. The nephrostomy tube was removed on postoperative day 5. Immediately thereafter, substantial bleeding occurred from the nephrostomy tract and through the ileal conduit. Although the patient remained hemodynamically stable, his hemoglobin level decreased from 10.4 to 9.4 g/dL. External compression of the nephrostomy site was applied, and a Foley catheter balloon was temporarily inflated within the ileal conduit to occlude the conduit outlet. Contrast-enhanced computed tomography revealed no active extravasation but demonstrated a hematoma within the right renal pelvis. Approximately 300 g of bloody output containing blood clots was observed from the ileal conduit. Given the severity of the bleeding and concern for continued blood loss, four units of packed red blood cells were transfused. Hemostasis was achieved without transcatheter arterial embolization. This case highlights that clinically significant hemorrhage may occur immediately after nephrostomy tube removal. Although no conclusions regarding the efficacy or safety of specific tube-removal techniques can be drawn from a single case, this experience underscores the importance of careful observation after tube removal, along with prompt evaluation and management if significant bleeding occurs.

## Introduction

Percutaneous nephrostomy (PCN) is an established procedure for the decompression of upper urinary tract obstructions caused by malignant diseases, urinary calculi, benign ureteral strictures, and other conditions [1-3]. Although PCN is generally considered safe, complications include infection, catheter-related complications, injury to adjacent organs, and hemorrhage [2-4]. Clinically, significant hemorrhage may require blood transfusion or transcatheter arterial embolization (TAE) [2,4]. Previous reports on PCN-related hemorrhagic complications have primarily focused on vascular injury during renal puncture. However, clinically significant hemorrhages triggered by nephrostomy tube removal have not been well described. Here, we report a case of clinically significant hemorrhage immediately after nephrostomy tube removal following radical cystectomy and ileal conduit urinary diversion.

## Case presentation

A 76-year-old man presented with asymptomatic gross hematuria. His medical history was significant for hypertension, and he was not taking antiplatelet or anticoagulant therapy. Magnetic resonance imaging suggested cT3a bladder cancer, and computed tomography (CT) revealed cN0M0 disease with bilateral hydronephrosis (Figure 1A). His serum creatinine level was 1.41 mg/dL and hemoglobin level was 12.7 g/dL. Transurethral resection of the bladder tumor was performed. Pathological examination revealed grade 3 urothelial carcinoma according to the 1973 World Health Organization (WHO) grading system, with invasion of the muscularis propria (at least pT2 according to the TNM classification) [5,6].

**Figure 1:**
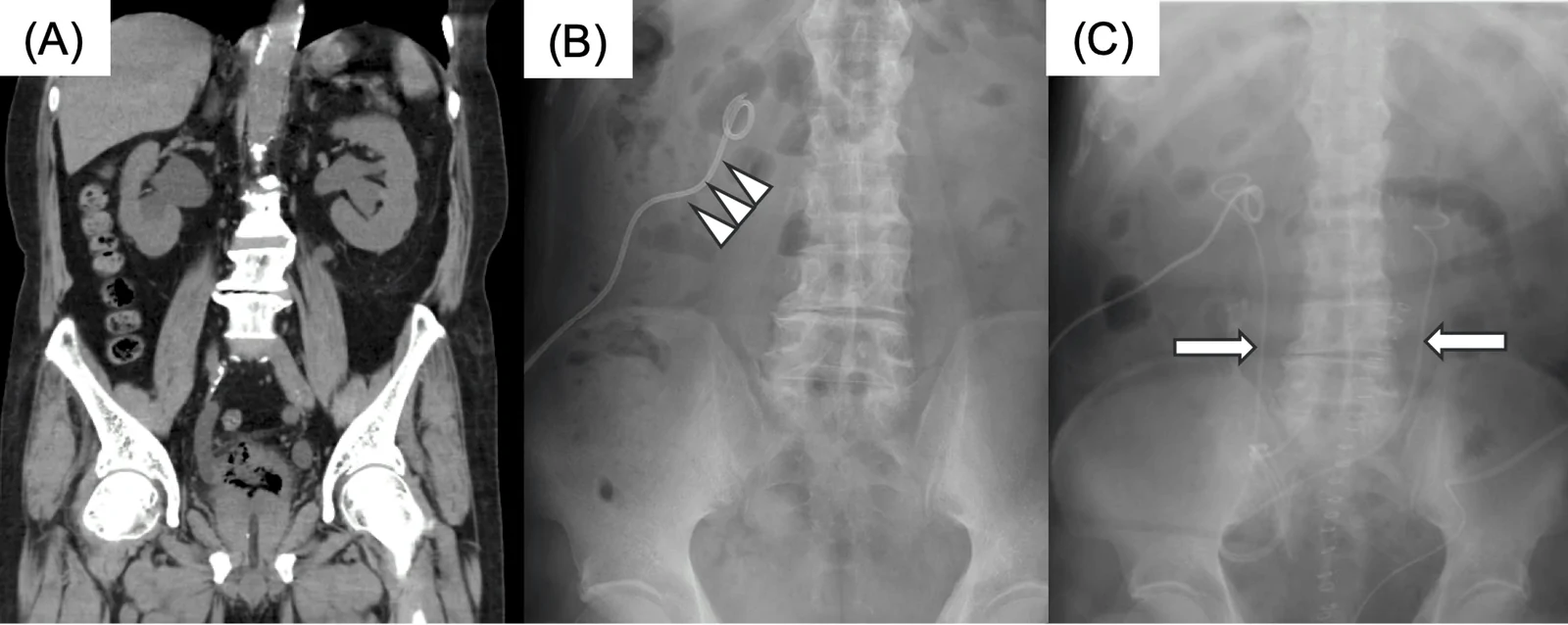
Preoperative imaging. (A) Preoperative computed tomography showing bilateral hydronephrosis. (B) White arrowheads indicate the right nephrostomy catheter. (C) White arrows indicate bilateral ureteral catheters.

He subsequently developed right obstructive pyelonephritis and underwent right PCN using an 8.3-Fr JINRO™ nephrostomy catheter (Boston Scientific, Marlborough, MA, USA) (Figure 1B). Nephrostomy was performed under ultrasound and fluoroscopic guidance, without Doppler imaging. A small amount of bleeding was observed through the puncture needle during access; however, a nephrostomy catheter was inserted as planned. Five days later, radical cystectomy, pelvic lymph node dissection, and ileal conduit urinary diversion were performed. The operative time was 261 min, and the estimated blood loss was 610 mL. Final pathological examination revealed urothelial carcinoma, pT3aN0. The nephrostomy tube was clamped after cystectomy (Figure 1C).

The nephrostomy tube was removed on postoperative day 5, corresponding to 10 days after PCN placement. Immediately after removal, bleeding occurred from the nephrostomy site, accompanied by a substantial bloody output with clots through the ileal conduit. Immediately before nephrostomy tube removal, his blood pressure was 159/75 mmHg and his heart rate was 82 beats/min. At the onset of bleeding, his blood pressure decreased to 124/65 mmHg and his heart rate increased to 96 beats/min. Despite these changes, his blood pressure remained within a stable range, although he appeared pale. His hemoglobin level had decreased from 12.7 g/dL at presentation to 10.4 g/dL on postoperative day 1 after cystectomy without transfusion and reached a nadir of 9.4 g/dL immediately after the acute bleeding episode (Table 1).

**Table 1:** Laboratory investigations. PT-INR, prothrombin time–international normalized ratio; aPTT, activated partial thromboplastin time.

Laboratory parameter	At presentation	Postoperative day 1 after radical cystectomy	Postoperative day 3 after radical cystectomy	Immediately after nephrostomy tube removal	Following day	Reference range
Hemoglobin (g/dL)	12.7	10.4	10.6	9.4	11.4	13.0–18.0
Serum creatinine (mg/dL)	1.41	0.86	0.85	0.91	0.95	0.65–1.10
Platelet count (×10³/µL)	341	215	217	196	203	150–450
PT-INR	0.93	—	—	0.98		0.8–1.1
aPTT (s)	24.9	—	—	24.8		25–35

A Foley catheter was inserted into the ileal conduit, and the balloon was inflated with 60 mL to temporarily occlude the conduit outlet (Video 1); this maneuver was not intended to directly compress the renal bleeding site. Approximately 300 g of bloody output containing blood clots was observed from the ileal conduit. Unenhanced CT demonstrated a hematoma within the right renal pelvis (Video 1), while early-phase contrast-enhanced CT showed no active contrast extravasation (Video 2). The balloon occlusion was released after 1 h, with no recurrent bleeding. Excretory-phase CT obtained after balloon removal continued to demonstrate the hematoma within the right renal pelvis (Video 3). 

**Video 1:** Unenhanced cine CT showing a hematoma in the right renal pelvis.

**Video 2:** Early-phase contrast-enhanced cine CT showing no active contrast extravasation.

**Video 3:** Excretory-phase cine CT obtained after removal of the Foley catheter balloon, showing a persistent hematoma in the right renal pelvis.

At the time of nephrostomy tube removal, the patient was not receiving antiplatelet or anticoagulant therapy. Mechanical venous thromboembolism prophylaxis consisted of elastic stockings and intraoperative intermittent pneumatic compression; pharmacologic prophylaxis was not administered. Immediately after the bleeding event, the platelet count was 196 × 10³/µL, PT-INR was 0.98, and aPTT was 24.8 s (Table 1). Given the substantial acute bleeding, superimposed on the perioperative decline in hemoglobin, and concern that continued or recurrent bleeding could result in further anemia and hemodynamic deterioration, four units of packed red blood cells were administered. The decision to transfuse was based on the overall clinical course and the amount of acute bleeding rather than the hemoglobin value alone. The bleeding subsequently ceased without further hemodynamic deterioration.

## Discussion

PCN is generally a safe procedure; however, hemorrhage remains an important complication. Quality improvement guidelines for PCN classify bleeding requiring transfusion or therapeutic intervention as a major complication [1,2]. In a prospective study, Wah et al. reported a high technical success rate for PCN, with serious hemorrhagic complications being relatively uncommon [3]. Waqas et al. reported bleeding requiring transfusion in 1.7% of patients after PCN [4]. However, these reports primarily addressed the complications related to PCN placement, and information regarding hemorrhage at the time of tube removal remains limited.

In the present case, Doppler imaging was not used during nephrostomy placement, and a small amount of bleeding was observed during puncture; therefore, injury to a small vessel at the time of PCN placement cannot be excluded. Avoiding vascular injury is important during percutaneous renal access, and color Doppler-guided puncture has been associated with reduced tract bleeding during percutaneous nephrolithotomy [7].

In this case, marked bleeding occurred immediately after removal of the nephrostomy tube. Early-phase contrast-enhanced CT showed no active contrast extravasation, and the bleeding source could not be identified. Possible mechanisms include bleeding from a small intrarenal vessel caused by injury to the nephrostomy tract, or loss of the local vascular tamponade effect provided by the catheter (Figure 2A). Mechanical irritation during the withdrawal of the curved pigtail portion may also have injured the renal parenchyma or nephrostomy tract; however, this mechanism remains speculative (Figure 2B).

**Figure 2:**
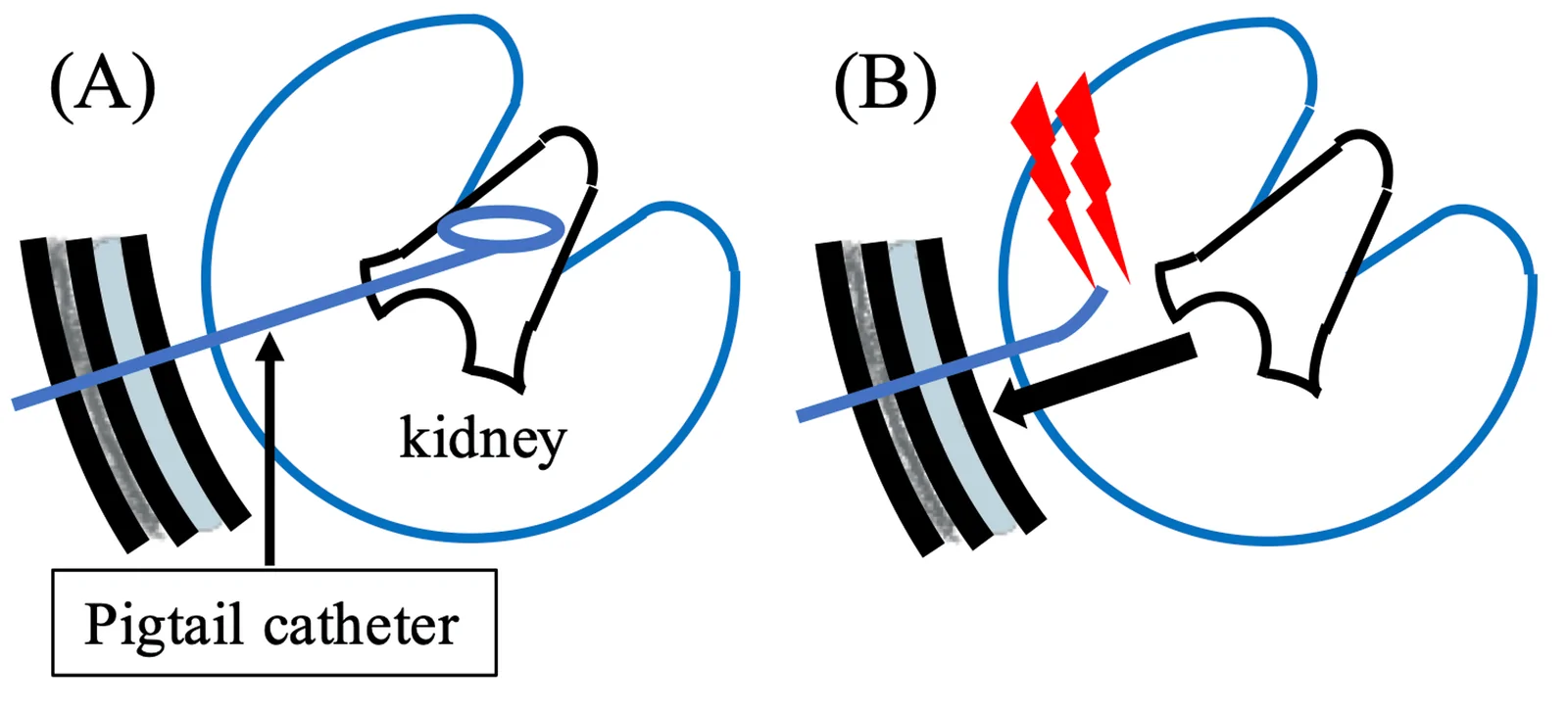
Proposed possible mechanisms of hemorrhage during nephrostomy tube removal. (A) A pigtail nephrostomy catheter was placed. (B) Schematic of the possible mechanical injury to the renal parenchyma or nephrostomy tract during catheter withdrawal. However, this mechanism was not directly demonstrated in this case. The schematic illustrations were created using Microsoft PowerPoint (Microsoft Corporation, Redmond, WA, USA).

The tube was removed 10 days after PCN placement, and incomplete tract maturation may have contributed to the bleeding; however, a causal relationship between catheter dwell time and hemorrhage cannot be established from a single case. If marked hematuria or bleeding from the nephrostomy site occurs after tube removal, the hemodynamic status should be promptly assessed, and contrast-enhanced CT should be considered to evaluate the extent of hemorrhage and the presence of active arterial bleeding. Persistent arterial bleeding or hemorrhage affecting hemodynamic stability should prompt the consideration of hemostatic interventions, including TAE. Although bleeding ceased after temporary balloon occlusion of the ileal conduit outlet, its causal hemostatic effect could not be established, and spontaneous hemostasis cannot be excluded. Nevertheless, this case highlights the potential for clinically significant hemorrhage following nephrostomy tube removal.

## Conclusions

Clinically significant hemorrhage may occur immediately after nephrostomy tube removal. When substantial bleeding or gross hematuria develops afterward, prompt clinical assessment and appropriate imaging should be considered based on clinical presentation. This single case does not establish the efficacy or safety of any specific tube-removal or hemostatic technique.
